# Representations underlying pronoun choice in Italian and English

**DOI:** 10.1177/17470218211051989

**Published:** 2021-10-22

**Authors:** Kumiko Fukumura, Coralie Hervé, Sandra Villata, Shi Zhang, Francesca Foppolo

**Affiliations:** 1 Division of Psychology, Faculty of Natural Sciences, University of Stirling, Stirling, UK; 2 Laboratoire FoReLLIS (EA3816), Université de Poitiers, France; 3 Department of Linguistics, University of Connecticut, Storrs, CT, USA; 4 Department of Psychology, University of Milano-Bicocca, Milan, Italy

**Keywords:** Language production, referential communication, similarity-based competition

## Abstract

Research has shown that speakers use fewer pronouns when the referential candidates are more similar and hence compete more strongly. Here we examined the locus of such an effect, investigating (1) whether pronoun use is affected by the referents’ competition at a non-linguistic level only (non-linguistic competition account) or whether it is also affected by competition arising from the antecedents’ similarities (linguistic competition account) and (2) the extent to which this depends on the type of pronoun. Speakers used Italian null pronouns and English pronouns less often (relative to full nouns) when the referential candidates compete more strongly situationally, while the antecedents’ semantic, grammatical or phonological similarity did not affect the rates of either pronouns, providing support for the non-linguistic competition account. However, unlike English pronouns, Italian null pronouns were unaffected by gender congruence between human referents, running counter to the gender effect for the use of non-gendered overt pronouns reported earlier. Hence, while both null and overt pronouns are sensitive to non-linguistic competition, what similarity affects non-linguistic competition partly depends on the type of pronouns.

Whenever referring to things or individuals, speakers have a choice of using pronouns (e.g., *she, it*) as opposed to names or nouns. A general assumption has been that speakers are more likely to choose less explicit referring expressions such as pronouns when the referent is more prominent or accessible in the discourse context (Ariel, 1990; Chafe, 1976; Givón, 1983; Grosz, Joshi, & Weinstein, 1995; Gundel, Hedberg, & Zacharski, 1993). For instance, speakers are more likely to use pronouns rather than nouns when the referent is mentioned as the grammatical subject than as other less prominent roles (Arnold, 2001; Brennan, 1995; Fukumura & Van Gompel, 2010, 2011, 2015; Rohde & Kehler, 2014; Stevenson, Crawley, & Kleinman, 1994). Research has also shown that speakers tend to use more pronouns when referring to human referents than when referring to non-human referents (Dahl & Fraurud, 1996; Fukumura & Van Gompel, 2011). An important question for theories of language production concerns the mechanisms that drive the speaker’s pronoun choice. Here we investigated the level of representation at which speakers choose to use a pronoun or noun and the extent to which this depends on the type of pronouns.

Specifically, research has shown that the choice of using a pronoun or noun is susceptible to similarity-based competition between the potential referents. Speakers of English use the pronoun “they” less often (as opposed to nouns) when two entities mentioned in the previous sentence are more similar because they have the same animacy (e.g., both are human; Fukumura & Van Gompel, 2011) or because they share the same sex (Arnold & Griffin, 2007; Fukumura, Hyönä, & Scholfield, 2013; Fukumura, Van Gompel, Harley, & Pickering 2011). Moreover, similarity-based competition has been shown to affect pronoun use when the similarity is based on the referents’ situational congruence: Speakers of English or Finnish are less likely to use a pronoun when the referential alternative is also compatible with the action carried out by the referent than otherwise (e.g., *He got off the horse* for a king when the referential alternative, a pilot, in the context is also on a horse and hence can get off the horse than otherwise). The referents’ situational attributes such as being on a horse are not expressed by the referring expressions. Hence, one possibility is that speakers choose whether to use a pronoun or noun at a non-linguistic level where speakers activate the referents’ non-linguistic features such as being a human, being a male, and being on a horse as retrieval cues of their message. When the referential candidates share one of these features, they compete more strongly for selection in the speaker’s message. Speakers then resolve this competition by activating more specific information about the referent, which in turn serves as input for more explicit referring expressions. We call this the *non-linguistic competition account*.

However, a hitherto-unexplored alternative possibility is that the referents’ similarities affect pronoun use because they affect linguistic competition. Pronoun production has been assumed to require access to the antecedent noun that the pronoun is replacing (Jescheniak, Schriefers, & Hantsch, 2001; Schmitt, Meyer, & Levelt, 1999). The choice of using a pronoun or noun could also be affected by competition between the antecedent nouns: Speakers use pronouns less often when the antecedent nouns of the referential candidates share similar meanings or lexical or phonological properties because speakers resolve lexical competition by activating the antecedent noun more strongly. Under this *linguistic competition account*, higher similarity between the referents, with respect to their gender, animacy, or situational attributes, results in fewer pronouns because these variables may affect lexical competition between the antecedent nouns, rather than competition between the referents at a non-linguistic level, contra the non-linguistic competition account.

Whether pronoun use is affected by the referents’ non-linguistic competition or the antecedents’ linguistic competition may depend on pronouns, however. While most previous pronoun production studies focused on “overt” pronouns, which are both phonetically and lexically realised, many languages such as Italian and Spanish allow “null” or “zero” pronouns that are not overtly realised. Although the preferences of null pronouns in the null pronoun languages have often been assumed to mirror those of (overt) pronouns in other languages such as English (Alonso-Ovalle, Fernández-Solera, Frazier, & Clifton, 2002; Carminati, 2002; Di Eugenio, 1998), the underlying representations might be different. Whereas the use of overt pronouns requires lexical access, starting from the activation of the antecedents’ conceptual or lexical properties such as person (e.g., you vs. she), animacy (e.g., she vs. it), number (e.g., she vs. they), and gender (e.g., she vs. he), access to such information may not be required for the use of null pronouns. Hence, while the referents’ non-linguistic competition might affect pronoun use regardless of whether the pronouns are overtly realised, lexical competition between the antecedent nouns may be more likely to affect pronoun use when the pronouns are overtly realised. Alternatively, the use of null pronouns may be affected by the antecedents’ similarity as well as the referents’ non-linguistic similarity, regardless of whether pronouns are overtly realised, in keeping with some linguistic theories, which assume that null pronouns carry the same information as overt pronouns and the antecedent nouns (D’Alessandro, 2015; Rizzi, 1982, 1986).

The overarching aim of this study was, therefore, to determine (1) whether the use of pronouns is affected by the similarity between the antecedent nouns, as well as the referents’ non-linguistic competition, and (2) whether different pronouns are differentially affected by similarity-based competition. To this end, we contrasted the effects of the referents’ non-linguistic competition with the effects of lexical competition on the use of Italian null pronouns (Experiments A) and English (overt) pronouns (Experiments B). Experiment 1 examined if speakers use fewer pronouns when the antecedents are semantically more related and thus compete more strongly or if pronoun use is only affected by the referents’ competition in the non-linguistic context. Experiment 2 then examined whether the pronoun rates are affected by the antecedents’ phonological similarity, as well as by the referents’ non-linguistic competition. Experiments 3 and 4 further examined whether the use of null pronouns is affected by the referents’ gender congruence. All experiments adopted a referential communication task, where speakers referred to the target referent for their addressee, who then had to identify the referent. We now report the experiments in turn.

## Experiment 1

A great deal of evidence suggests that nouns’ semantic category congruence affects lexical competition; word substitution errors occur more frequently when the substituted words are semantically related (Dell & Reich, 1981; Fromkin, 1973; Garrett, 1975) than when they are not. Research has also demonstrated that speakers are slower in picture naming when they hear or see a distractor word that is semantically related to the to-be-named picture (e.g., dog–cat) than when the distractor word is unrelated (e.g., dog–car; Glaser & Düngelhoff, 1984; Glaser & Glaser, 1989; La Heij, 1988). Interestingly, although semantic category congruence results from the shared words’ meaning or lexical concept (Levelt, Roelofs, & Meyer, 1999), it has been thought to affect competition at the lexical level; the referents’ semantic category congruence does not hamper a non-linguistic task that does not involve naming (Schriefers, Meyer, & Levelt, 1990). On pronoun production, Jescheniak et al. (2001) reported delayed pronoun production in German when speakers heard a distractor word that was semantically related to the target (e.g., *jacket* for *coat*) relative to when the distractor word was unrelated. In their study, participants were asked to produce pronouns only. Here we asked whether the choice of using a null pronoun in Italian (Experiment 1A) or the pronoun “it” in English (Experiment 1B) relative to repeated nouns is affected by the referents’ semantic category congruence or whether pronoun use is affected by the referents’ non-linguistic competition only.

**Figure 1.**
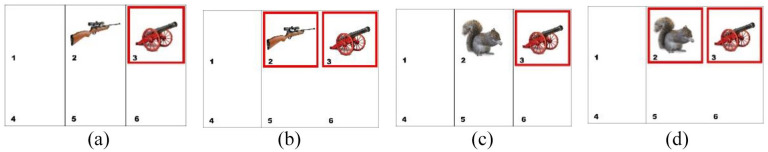
Example visual display (Experiment 1B). Semantically related: (a) one-box and (b) two-box condition. Semantically unrelated: (c) one-box and (d) two-box condition.

In a referential communication task, participants first read aloud the context sentence (e.g., 1a) to their addressee (experimental confederate), which introduced two referential candidates in the visual display, as shown in Figure 1. Then, one of the objects, the “target object” hereafter, moved to another location (e.g., the cannon moves to Number 6), while the other object, the “referential competitor” hereafter, remained still. The task of the participant was to describe this change (2c), starting with the adverbial *Adesso* or *Now*. We made two manipulations. First, the referential candidates were taken from either the same categories (semantically related condition; Figure 1a and b), or different semantic categories (semantically unrelated condition; Figure 1c and d). Second, the referential candidates were either situationally congruent or incongruent; In the situation-incongruent (one-box) condition, only the target referent was in a red box, which indicated to participants that only that entity could move, whereas in the situation-congruent (two-box) condition (Figure 1b and d), the two entities were both in a red box.

**Table:** 

Experiment 1A: Italian	Experiment 1B: English
1. Context sentences	
(a) Il cannone accanto al fucile è sul numero 3.	The cannon next to the rifle is on Number 3.
(b) Il cannone accanto al leone è sul numero 3.	The cannon next to the squirrel is on Number 3.^1^
2. Target descriptions	Now {the cannon/it} is on Number 6.
(c) Adesso {il cannone / Ø} è sul numero 6.	

We recorded two measures: the choice of referring expressions and the onsets of repeated nouns following *Adesso*/*Now* in the target descriptions. If semantic category congruence affects lexical competition between the antecedent nouns, speakers should be slower when they repeat the antecedent nouns (i.e., when they produce repeated nouns) in the semantically related condition than in the semantically unrelated conditions. Crucially, the non-linguistic competition account assumes that speakers decide whether to use a pronoun or noun at a non-linguistic level, without accessing the antecedent’s lexical information. Hence, assuming that semantic category congruence affects lexical competition rather than non-linguistic competition (cf. Schriefers et al., 1990), the referents’ semantic category congruence should not affect the rates of pronouns. Instead, the pronoun rates should only be affected by the referents’ situational congruence; we should observe fewer pronouns in the two-box context than in the one-box context. By contrast, the linguistic competition account predicts that speakers should use fewer pronouns when the antecedents are semantically related than when they are semantically unrelated, because pronoun use is sensitive to competition between the antecedent nouns.

### Method

#### Participants

A total of 32 native speakers of Italian and 32 native speakers of British English were recruited from the University of Milano-Bicocca and the University of Strathclyde student community, respectively, in exchange of course credits or cash. They reported to be aged between 18 and 30 years, with no visual impairment.

#### Materials

We constructed 48 experimental items (see supplemental materials). Each item comprised a 2 × 3 display containing two objects (Figure 1) and a context sentence (1a and 1b), which introduced the objects linguistically. The target was mentioned in the subject position and the competitor in the prepositional phrase modifying the target. The spatial relations of the objects in the display were described by one of the following three phrases: *next to*, *above*, or *below*. In the display, the target and the competitor were either both in a red box (two-box; situation-congruent condition) or only the target was in a red box (one-box; situation-incongruent condition). The objects were either from the same semantic category (semantically related condition) or from different categories (semantically unrelated condition). The same target objects and related competitor were used for Italian and English experiments, except for four competitor objects (*dove* for Italian and *duck* for English, *sunflower* for Italian and *rose* for English, *potato* for Italian and *tomato* for English, and *bench* for Italian and *table* for English) and one target object (*vase* for Italian and *beer glass* for English). Each competitor occurred in all four conditions across different items, counterbalancing the properties of the competitors across conditions. The referential candidates always had the same grammatical gender in Italian, and the target and competitor were phonologically dissimilar. Because of these constraints, different competitors were assigned to the unrelated competitor condition in Italian and English. In addition, 65 filler items were constructed, where we varied the position of the target character in the sentences as well as the number of entities or boxes in the display.

#### Design

The competitor was semantically related or semantically unrelated to the target and it was either in the same situation as the target (both in the red box) or in a different situation from the target (it was not in the box). Experiment 1A was conducted in Italian and Experiment 1B was done in English. In addition, each experiment was divided into two blocks and we counterbalanced the order in which the two blocks were presented. Thus, each experiment employed a 2 (Semantic relation: related vs. unrelated) × (Situational congruence: congruent vs. incongruent) × 2 (Block order: Block 1 first vs. Block 2 first) repeated measures design, resulting in the creation of eight lists (block order was not included in the analyses as this was merely a counterbalancing variable). Together with the 65 filler items, the 48 experimental items were distributed across the eight lists, each list having six experimental items in each condition, with one version of each item. The items of each list were presented in a fixed quasi-random order, subject to the constraint that objects of similar category types do not appear consecutively. In each experiment, four participants were randomly assigned to each list (with 32 participants taking part in each experiment).

#### Procedure

Each session involved a naive participant and an experimental confederate. The participant took part as the speaker and the confederate as the addressee. The confederate was treated as a naive participant throughout, and no participants indicated in a post-experimental questionnaire that they thought the confederate was not genuine. Both participants sat side-by-side at a table, each facing a computer screen. The experimenter explained the tasks, which was followed by five practice trials. On each trial, both participants were presented with a 2 × 3 display on their monitor (Figure 1). Participants then pressed a key, which then triggered a presentation of a context sentence (1), appearing below the display. On the addressee’s screen, the same display was also shown but not the sentence. The addressee could not see the participant’s screen, but participants were able to see the addressee’s screen. Participants then read aloud the sentence for the addressee and then pressed a key. This triggered the change of the target’s position in the display, as well as the presentation of a beep, which was used to determine the timing of the target display in the analyses of the onset latencies.

At the instructions, the experimenter explained to participants that a red box indicated which object was going to move: If two objects are in a red box, one of them was going to move. Participants were asked to describe the change in the display (e.g., *Now the cannon/it is on Number 3*), such that the addressee was able to point at the target object and its new location. Participants were asked to produce their response starting with *Adesso*/*Now*, so they would produce a new sentence. The experimenter provided example descriptions during the instructions using pronouns and repeated nouns (“for instance, you could say *Now, he is on Number 6* or *Now the man is on Number 6*”) and told participants that they were free to vary the choice of expression. This was done to discourage participants from strategically using the same type of referring expressions throughout. The addressee was asked to point at the object and its new location to the speaker. To ensure that speakers paid attention to the addressee’s comprehension, speakers were asked to press a “yes” key if they thought that their addressee understood what they meant and a “no” key if they thought otherwise. We used E-Prime to present the stimuli and record participants’ speech. Each session took approximately 30–40 min. The ethical approaches of the experiments reported in this article were approved by the relevant institutional review boards, and written informed consent was obtained from all participants.

#### Scoring

We scored whether participants produced null pronouns (*n* *=* 621) or repeated nouns (*n* *=* 835) in Italian (Experiment 1A) and pronouns (*n* *=* 674) or repeated nouns (*n* *=* 826) in English (Experiment 1B). Participants only produced a limited number of overt pronouns (*n* *=* 58) in Italian, presumably because the referent was always the grammatical subject and null pronouns are generally more preferred for the subject antecedent than overt pronouns in Italian (Carminati, 2002; Di Eugenio, 1998). Moreover, the most frequent lexical pronouns in Italian are *lei* and *lui* (corresponding to she and he in English), but these are typically used for human referents only, and the counterpart of these pronouns for inanimate referents (i.e., *essa*, *esso*) are rarely used in speech (Di Eugenio, 1998). These cases were thus excluded from analyses. Other excluded cases include those where participants made substitution errors by referring to the competitor (e.g., *Now the saw moved to 2 . . . no, the axe moved to 2*; *n* *=* 7 in Experiment 1A; *n* *=* 1 in Experiment 1B); participants accidentally skipped the trials (*n* *=* 4 in Experiment 1A, *n* *=* 7 in Experiment 1B); they altered their initial choice of expressions (e.g., *Now it’s . . . the pigeons on Number 2; Now the . . . now it’s moved to Number 6*; *n* *=* 5 in Experiment 1A; *n* *=* 3 in Experiment 1B). In addition, we excluded trials where participants used a non-repeated noun (*n* *=* 1 in Experiment 1A; *n* *=* 1 in Experiment 1B) or they used demonstrative pronouns in Italian (*n* *=* 5 in Experiment 1A) or dropped subjects in English (*n* *=* 25 in Experiment 1A). In total, 5.2% (*n* *=* 80) and 2.3% (*n* *=* 36) of trials were excluded from analyses for Experiment 1A and Experiment 1B, respectively.

### Results

#### Choice of referring expressions

**Figure 2.**
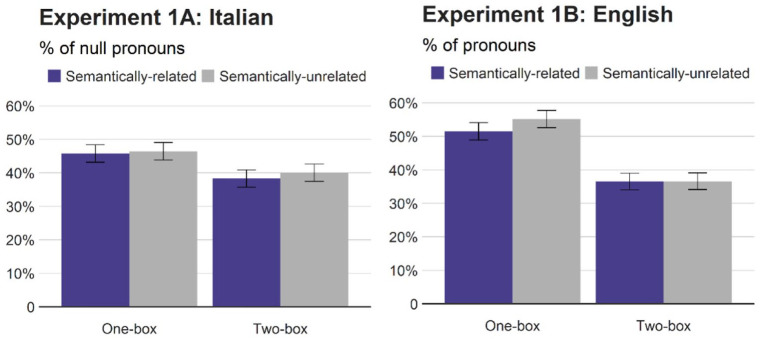
Percentages of null pronouns (Italian) and pronouns (English) relative to repeated nouns (Experiment 1). Error bars represent standard errors.

Figure 2 reports the percentages of null pronouns (relative to repeated nouns) in Italian (Experiment 1A) and the percentages of pronouns relative to repeated nouns in English (Experiment 1B). As detailed in the scoring section, there were only a limited number of overt pronouns in Italian. Hence, we analysed the rates of null pronouns relative to repeated nouns in Italian and the rates of the pronoun “it” in English relative to repeated nouns using logit mixed effects modelling (e.g., Baayen, Davidson, & Bates, 2008), using the lme4 package (Bates, Mächler, Bolker, & Walker, 2015) in R (R Core Team, 2019). The initial analyses included by-participants and by-items random intercepts as well as random slopes for all the relevant fixed factors (i.e., situational congruence, semantic category congruence, and the interaction between the two for Experiment 1; Barr, Levy, Scheepers, & Tily, 2013). We avoided potential overparameterisation by suppressing correlations between random effects (Bates, Kliegl, Vasishth, & Baayen, 2015; Bates et al., 2015; Kliegl, 2014; Singmann & Kellen, 2020). We then removed random slopes with zero or close-to-zero variances to mitigate potential singularity, though this procedure did not alter the results from the full model. The fixed factors were mean-centred and standardised: Similar to sum coding, centering reduces collinearity between variables allowing us to interpret the results in terms of main effects and interactions (Baayen et al., 2008), and it also facilitates convergence in R (Gelman & Hill, 2007). Table 1 summarises the results.

**Table 1.** Analyses on the choice of expressions in Experiment 1.

Effects	Estimate	*SE*	*z*	*p*
Italian (Experiment 1A)				
(Intercept)	−0.97	0.61	−1.60	.111
Situational congruence	−0.34	0.15	−2.26	.024*
Semantic category congruence	0.08	0.09	0.91	.362
Situational × Semantic category congruence	0.02	0.09	0.21	.836
English (Experiment 1B)				
(Intercept)	−0.49	0.61	−0.80	.423
Situational congruence	−0.69	0.13	−5.45	<.001*
Semantic category congruence	0.09	0.08	1.23	.219
Situational × Semantic category congruence	−0.09	0.08	−1.25	.211
Combined analysis				
(Intercept)	−0.73	0.43	−1.68	.093
Language	−0.24	0.43	−0.56	.574
Situational congruence	−0.52	0.10	−5.37	<.001*
Semantic category congruence	0.08	0.06	1.48	.138
Language × Situational congruence	0.18	0.10	1.80	.071
Language × Semantic category congruence	−0.01	0.06	−0.18	.854
Situational × Semantic category congruence	−0.04	0.06	−0.62	.534
Language × Situational congruence × Semantic category congruence	0.06	0.06	1.04	.301

*SE*: standard error. *Significance level α = 0.05.

The analyses on Italian null pronouns revealed a significant main effect of situational congruence (box manipulation), indicating fewer null pronouns (i.e., more repeated nouns) in the two-box context (39%) than in the one-box context (46%). However, neither semantic category congruence nor the situational congruence × semantic category congruence was significant. The analyses on English pronouns showed the same pattern of the results. Speakers of English produced fewer pronouns in the two-box context (37%) than in the one-box context (53%), but their pronoun usage was unaffected by the referents’ semantic category congruence nor the interaction between the two variables. The combined analyses including language (Italian vs. English), situational congruence, and semantic category congruence showed no significant effect of language, indicating that the rates of Italian null pronouns and English pronouns did not differ reliably. There was a marginal interaction between situational congruence and language, reflecting a slightly larger effect of situational congruence in English than in Italian. Critically, the main effect of semantic category congruence was non-significant and there was no language × semantic category congruence interaction. Although the combined analyses were based on the data from 64 participants, the Bayes factor for semantic category congruence, estimated from the Bayesian Information Criterion values (Wagenmakers, 2007), was as low as 0.054, providing strong support for the null hypothesis.

#### Onset latencies

We then examined whether the referents’ situational and semantic similarities affected processing times, particularly the onset latencies of the repeated nouns following the onsets of *Adesso/*Now*.* The onsets of the target display (i.e., a beep presented simultaneously with the target display), *Adesso*/*Now*, and the repeated nouns were identified using CheckVocal (Protopapas, 2007). We computed the times taken by participants to trigger the presentation of the target display, the initiation times for *Adesso*/*Now* (after the onset of the target display) and for the repeated nouns (after *Adesso*/*Now*). We assumed the latencies for the target display as reflecting the times needed for participants to process the context (i.e., the context sentence and the initial display), the latencies for *Adesso*/*Now* as representing the times needed to apprehend the target display and the latencies for the repeated nouns as reflecting the planning times for the repeated nouns. The latencies were analysed sequentially, starting from the latencies for the target display for cases where participants produced repeated nouns or pronouns, followed by the latencies for *Adesso*/*Now* and then the latencies for repeated nouns, with extreme reaction times whose *z*-scores exceeded 3.29 removed from each analysis, together with cases where the onset could not be identified. For the analyses on the initiation times for *Adesso*/*Now* and those for repeated nouns, we further excluded cases where participants did not produce *Adesso*/*Now* or placed *Adesso*/*Now* in a non-initial position, or disfluencies occurred around the nouns. Table 2 reports the mean onset latencies for the target display (Experiment 1A: *n* = 1,444; Experiment 1B: *n* = 1,473), *Adesso*/*Now* (Experiment 1A: *n* = 1,350; Experiment 1B: *n* = 1,226) and repeated nouns (Experiment 1A: *n* = 741; Experiment 1B: *n* = 696).

**Table 2.** Mean onset latencies (in milliseconds) in Experiment 1.

Situational congruence	Semantic relation	Target display	*Adesso*/*Now*	Repeated noun
Italian (Experiment 1A)				
One-box	Related	3,826 (42)	882 (16)	443 (13)
	Unrelated	3,765 (40)	848 (18)	420 (10)
Two-box	Related	3,979 (42)	912 (20)	433 (11)
	Unrelated	3,877 (41)	904 (19)	421 (11)
English (Experiment 1B)				
One-box	Related	3,579 (37)	836 (17)	364 (10)
	Unrelated	3,566 (41)	829 (16)	350 (10)
Two-box	Related	3,694 (41)	838 (17)	373 (9)
	Unrelated	3,792 (44)	863 (17)	346 (7)

Numbers in brackets represent standard errors.

We carried out linear mixed effects analyses (Baayen et al., 2008) on the log-transformed onset latencies. Table 3 summarises the results. The analyses of the Italian data showed the delayed production of repeated nouns in the same semantic category condition (438 ms) than in the different semantic category condition (420 ms), while the referents’ situational congruence (box manipulation) had no effect on the latencies of the repeated nouns. The analyses of the English data showed similar results with slower repeated noun production latencies in the same semantic category condition (369 ms) than in the different category condition (348 ms), whereas no effect of situational congruence nor interaction were found on the latencies of the repeated nouns. In both languages, the referents’ situational congruence delayed the presentation of the target display, indicating that participants were slower in processing the context (i.e., the sentence and the initial display) in the two-box condition (Italian: 3,928 ms; English: 3,742 ms) than in the one-box condition (Italian: 3,796 ms; English: 3,572 ms). The initiation of their verbal responses was also delayed in Italian, with slower latencies for *Adesso*/*Now* in the two-box context (908 ms) than in the one-box context (865 ms), and the initiation times were also marginally affected in English.

**Table 3.** Analyses on onset latencies in Experiment 1.

Onset	Effect	Estimate	*SE*	*t*	*p*
Italian (Experiment 1A)					
Target display	Situational congruence	0.018	0.004	4.46	<.001*
	Semantic category congruence	−0.011	0.004	−2.67	.008*
	Situational × Semantic congruence	−0.005	0.004	−1.14	.257
*Adesso*	Situational congruence	0.019	0.009	2.06	.040*
	Semantic category congruence	−0.021	0.010	−2.10	.045*
	Situational × Semantic congruence	0.005	0.009	0.60	.547
Repeated noun	Situational congruence	−0.001	0.012	−0.07	.948
	Semantic category congruence	−0.022	0.010	−2.19	.042*
	Situational × Semantic congruence	−0.002	0.011	−0.17	.868
English (Experiment 1B)					
Target display	Situational congruence	0.022	0.004	5.93	<.001*
	Semantic category congruence	0.004	0.005	0.81	.425
	Situational × Semantic congruence	0.008	0.005	1.62	.117
*Now*	Situational congruence	0.014	0.008	1.80	.072
	Semantic category congruence	0.005	0.008	0.62	.535
	Situational × Semantic congruence	0.010	0.008	1.34	.182
Repeated noun	Situational congruence	−0.001	0.009	−0.14	.890
	Semantic congruence	−0.027	0.011	−2.53	.015*
	Situational × Semantic congruence	−0.005	0.010	−0.53	.606

*SE*: standard error. *Significance level α = 0.05.

#### Discussion

The referents’ situational congruence in the non-linguistic context lowered the rates of both Italian null pronouns and English pronouns, but it had no effect on the onset latencies of the repeated nouns. Conversely, the referents’ semantic category congruence did not affect pronoun use but it delayed the onset latencies for the repeated nouns. That is, the referents’ semantic category congruence that delayed the production of the repeated nouns did not affect pronoun use, whereas the referents’ situational congruence that did not delay the production of the repeated nouns reduced the rates of both Italian null pronouns and English pronouns. The delayed production of the repeated nouns following the referents’ semantic congruence was in keeping with research that showed that the distractor word’s semantic category congruence to the target word affects lexical competition, delaying the production of the target word. Critically, this did not affect the choice of using a pronoun or a noun, unlike the referents’ competition in the non-linguistic context. Hence, the findings provided support for the non-linguistic competition account, which predicts that lexical competition between the antecedent nouns does not affect pronoun choice, because speakers choose whether to use a pronoun or noun at a non-linguistic level. The results also echo the finding that the referents’ semantic category congruence causes competition at the lexical level, but not at a non-linguistic level (Damian & Bowers, 2003; Schriefers et al., 1990) and underscore the distinction between the non-linguistic/conceptual information and the semantic information.

## Experiment 2

Experiment 1 showed that the antecedents’ semantic category congruence does not affect pronoun use. Experiment 2 examined whether the antecedents’ phonological similarity results in fewer pronouns. Similar to semantic similarity, phonological similarity is known to affect the likelihood of substitution errors (Dell & Reich, 1981; Fay & Cutler, 1977; Fromkin, 1973; Griffin & Wangerman, 2013; Nooteboom, 1973), though phonologically related distractors speed up naming (Posnansky & Rayner, 1977; Rayner & Springer, 1986). There is some evidence indicating that pronoun production involves access to the antecedent’s phonology. In the study by Schmitt et al. (1999), in response to a lead-in sentence such as *Die Blume ist rot* (The flower is red), speakers of German produced a pronoun, as in *Sie wird blau* (It turns blue). Before producing the pronoun, participants carried out a lexical decision to an auditory distractor word. Lexical decision times were delayed when the distractor was phonologically related to the antecedent noun (e.g., *Bluse*, blouse, for *Blume*, flower) than when it was unrelated (*Kelle*, ladle). However, Jescheniak et al. (2001) found that the antecedent’s phonological information is inactive during pronoun production: In their study, the distractor word’s phonological similarity to the target had no effect on the onset latencies of the pronouns.

Experiment 2 examined whether the choice of using a pronoun or a noun is affected by the antecedents’ phonological similarity because phonologically related competitors enhance competition at the lexical level (cf. Cutting & Ferreira, 1999; Dell, 1986; Ferreira & Griffin, 2003; but see also Levelt et al., 1999). The design was parallel to the one in Experiment 1. The antecedents’ semantic similarity was replaced with their phonological similarity while the referents’ situational congruence was manipulated as in Experiment 1. The referential competitor either shared the same initial phoneme (3a) or they had different phonemes (3b). If speakers use fewer pronouns when the antecedent nouns are phonologically similar than when they are not, this will indicate that pronoun use is sensitive to competition arising from phonological similarity between the antecedent nouns, in support of the linguistic competition account. Alternatively, pronoun choice may be affected by the referents’ situational congruence, but not by the antecedents’ phonological similarity; speakers may choose whether to use a pronoun or a noun at a non-linguistic level, producing fewer pronouns in the two-box context than in the one-box context, without accessing the antecedents’ phonology, as claimed by the non-linguistic competition account.

**Table:** 

Experiment 2A: Italian	Experiment 2B: English
3. Context sentences	
(a) Il cannone accanto al calzino è sul numero 3.	The cannon next to the cactus is on Number 3.
(b) Il cannone accanto al rubino è sul numero 3.	The cannon next to the balloon is on Number 3.
4. Target descriptions	
(c) Adesso {il cannone / Ø} è sul numero 6.	Now {the cannon/it} is on Number 6.

### Method

#### Participants

A total of 32 Italian native speakers (Experiment 2A) and 32 English native speakers (Experiment 2B) were recruited from the same population as before.

#### Materials and procedure

**Figure 3.**
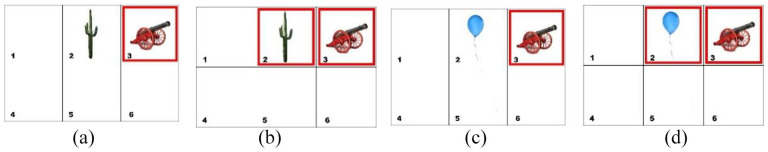
Example displays from Experiment 2B. Phonologically related: (a) one-box and (b) two-box condition. Phonologically unrelated: (c) one-box and (d) two-box condition.

Target referents were the same as in Experiment 1. We manipulated the competitor’s phonological similarity to the target by having a new set of competitor objects for each language. In the phonologically related condition (3a), the competitor object had the same initial consonant and similar vowels as the target object, whereas in the phonologically unrelated condition (3b), the competitor had different initial phonemes from the target. Within each phonological condition, both the referent and the competitor were either both in a red box (two-box condition, Figure 3b and d) or only the referent was in a red box (one-box condition, Figure 3a and c). The competitors were always semantically unrelated to the target and they had the same grammatical gender in Italian. Each competitor occurred in all conditions across different items, counterbalancing competitors across different conditions.

#### Design

We used a 2 (phonological relatedness: related vs. unrelated) × 2 (situational congruence: one-box vs. two-box) × 2 (block order: Block 1 first vs. Block 2 first) repeated design for each experiment, which was tested in Italian (Experiment 2A) and English (Experiment 2B). Across eight lists, 48 experimental items and 65 filler items (same as before) were distributed as before.

#### Scoring

We scored whether participants produced null pronouns (*n* *=* 651) or repeated nouns (*n* *=* 864) in Italian (Experiment 2A) or they used pronouns (*n* *=* 610) or repeated nouns (*n* *=* 906) in English (Experiment 1B). We excluded cases with substitution errors (*n* *=* 8 in Experiment 2A; *n* *=* 6 in Experiment 1B), changed responses (*n* *=* 1 in Experiment 2A; *n* *=* 2 in Experiment 1B), and technical errors (*n* *=* 5 in Experiment 2A; *n* *=* 5 in Experiment 1B). In addition, cases with overt pronouns in Italian (*n* *=* 2 in Experiment 2A) or dropped verbs after nouns in Italian (*n* *=* 5 in Experiment 2A) and cases with omitted subjects in English (*n* *=* 7 in Experiment 1B) were excluded. In total, 21 trials (1.4%) from Experiment 2A and 20 trials (1.3%) from Experiment 2B were excluded, respectively.

### Results

#### Choice of referring expressions

**Figure 4.**
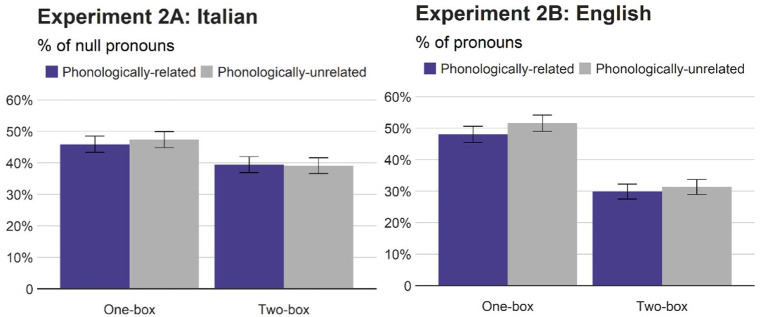
Percentages of null pronouns (Italian) and pronouns (English) relative to repeated nouns (Experiment 2). Error bars represent standard errors.

Figure 4 reports the mean percentages of null pronouns in Italian and pronouns in English (relative to repeated nouns). The analyses were carried out as before. Table 4 summarises the results. The analysis on the Italian null pronouns revealed a main effect of situational congruence, with more null pronouns (i.e., fewer repeated nouns) in the one-box condition (47%) than in the two-box condition (39%), whereas no significant main effect of phonological relation nor a significant interaction were found. Similarly, speakers of English produced more pronouns in the one-box condition (50%) than in the two-box condition (31%), but neither phonological similarity nor the interaction had an effect. The combined analysis found no evidence that the referents’ situational congruence affected Italian null pronouns and English pronouns differently, and the Bayes factor for phonological similarity in the combined analysis was as low as 0.090.

**Table 4.** Analyses on the choice of expressions in Experiment 2.

Effects	Estimate	*SE*	*z*	*p*
Italian (Experiment 2A)				
(Intercept)	−1.75	1.31	−1.33	.183
Situational congruence	−0.85	0.25	−3.35	.001*
Phonological relation	0.09	0.12	0.70	.483
Situational × Phonological relation	−0.07	0.15	−0.43	.668
English (Experiment 2B)				
(Intercept)	−1.24	0.68	−1.83	.067
Situational congruence	−0.88	0.18	−4.82	<.001*
Phonological relation	0.13	0.08	1.67	.096
Situational × Phonological relation	−0.04	0.08	−0.50	.617
Combined analysis				
(Intercept)	−1.37	0.62	−2.20	.028*
Language	0.05	0.62	0.08	.935
Situational congruence	−0.82	0.14	−5.84	<.001*
Phonological relation	0.11	0.07	1.61	.108
Language × Situational congruence	0.07	0.14	0.53	.593
Language × Phonological relation	−0.03	0.07	−0.41	.681
Situational × Phonological relation	−0.05	0.07	−0.74	.461
Language × Situational × Phonological relation	−0.01	0.07	−0.18	.861

*SE*: standard error. *Significance level α = 0.05.

#### Onset latencies

As before, the log-transformed reaction times were analysed for the presentation of the target display (Experiment 2A: *n* = 1,504; Experiment 2B: *n* = 1,481), the onset of *Adesso*/*Now* (Experiment 2A: *n* = 1,373; Experiment 2B: *n* = 1,352) and the onset of the repeated nouns (Experiment 2A: *n* = 729; Experiment 2B: *n* = 790). Table 5 reports the means and Table 6 summarises the results. Unlike semantic category congruence, phonological similarity did not affect the onset latencies of the repeated nouns in Italian nor English. As in Experiment 1, situational congruence delayed the onset of the target display in both Italian and English; participants spent longer time processing the context (i.e., the initial display and the context sentence) in the two-box condition (Italian: 4,011 ms; English: 3748 ms) than in the one-box condition (Italian: 4,171 ms; English: 3,643 ms). In the English experiment, participants were also slower in starting to produce their verbal responses in the two-box context (783 ms) than in the one-box context (750 ms).

**Table 5.** Mean onset latencies (in milliseconds) in Experiment 2.

Situational congruence	Phonological relation	Target display	*Adesso*/*Now*	Repeated noun
Italian (Experiment 2A)				
One-box	Related	4,046 (47)	773 (13)	431 (11)
	Unrelated	3,976 (42)	777 (13)	425 (10)
Two-box	Related	4,197 (48)	797 (15)	436 (11)
	Unrelated	4,146 (46)	788 (15)	429 (10)
English (Experiment 2B)				
One-box	Related	3,668 (34)	757 (14)	354 (10)
	Unrelated	3,618 (34)	743 (14)	358 (10)
Two-box	Related	3,778 (40)	782 (15)	355 (8)
	Unrelated	3,719 (39)	783 (14)	346 (8)

Numbers in brackets represent standard errors.

**Table 6.** Analyses on onset latencies in Experiment 2.

Onset	Effect	Estimate	*SE*	*t*	*p*
Italian (Experiment 2A)					
Target display	Situational congruence	0.019	0.004	4.39	<.001*
	Phonological relation	−0.006	0.004	−1.61	.115
	Situational × Phonological relation	0.002	0.004	0.40	.690
*Adesso*	Situational congruence	0.005	0.007	0.77	.447
	Phonological relation	< 0.001	0.007	−0.01	.989
	Situational × Phonological relation	−0.005	0.007	−0.70	.482
Repeated noun	Situational congruence	0.004	0.008	0.51	.612
	Phonological relation	−0.012	0.009	−1.29	.216
	Situational × Phonological relation	−0.001	0.008	−0.14	.887
English (Experiment 2B)					
Target display	Situational congruence	0.013	0.004	3.33	.002*
	Phonological relation	−0.007	0.004	−1.82	.075
	Situational × Phonological relation	< 0.001	0.004	0.07	.946
*Now*	Situational congruence	0.027	0.008	3.20	.002*
	Phonological relation	−0.006	0.008	−0.72	.472
	Situational × Phonological relation	0.010	0.008	1.30	.195
Repeated noun	Situational congruence	−0.007	0.008	−0.84	.399
	Phonological relation	−0.001	0.008	−0.16	.870
	Situational × Phonological relation	−0.013	0.011	−1.17	.258

*SE*: standard error. *Significance level α = 0.05.

### Discussion

As in Experiment 1, the referents’ situational congruence led to fewer Italian null pronouns and English pronouns, whereas the antecedents’ phonological similarity did not. These findings provided further support to the non-linguistic competition account; the choice of using a pronoun or a noun is affected by the referents’ competition at a non-linguistic level only; it is unaffected by the similarity between the antecedent nouns. Phonological similarity between the antecedent nouns did not affect the onset latencies of repeated nouns. One reason for this may be that the nouns were preceded by the determiner, which may have neutralised the phonological similarity manipulated on the initial phonemes of the nouns. Importantly, the absence of a phonological similarity effect on the onset latencies cannot be responsible for the lack of a phonological similarity effect on pronoun use; the referents’ situational congruence led to fewer pronouns, but it did not delay the production of repeated nouns.

In this study, phonologically similar words were matched on the initial phonemes, as in other studies (Cleland & Pickering, 2003; Damian & Martin, 1999). One may, however, ask if pronoun choice and the production of repeated nouns could be affected by the antecedents’ phonological similarity with a stronger similarity manipulation. Findings in French, where the antecedents’ phonological similarity was matched on the initial syllables, showed no evidence that the use of pronouns is affected by the antecedents’ phonological similarity (Fukumura, Pozniak, & Alario, 2020). Moreover, a stronger phonological similarity will increase the temporal ambiguity of the repeated nouns. Ferreira, Slevc, & Rogers (2005) showed that speakers are often insensitive to the ambiguity that arises from homophony (bat in the context of a flying bat and a baseball bat). They argued that speakers do not avoid linguistic ambiguity that arises from homophony, because speakers avoid referential ambiguity based on non-linguistic similarity, and phonological similarity cannot be represented at a non-linguistic level, where speakers choose referring expressions. Hence, a stronger antecedent phonological similarity is unlikely to alter the pattern of the current results.

## Experiment 3

Experiments 1 and 2 showed that not only English pronouns but also Italian null pronouns are sensitive to the referents’ situational congruence. But this does not guarantee that the use of null pronouns is sensitive to the referents’ similarity per se. Specifically, gender congruence between human referents has been found to reduce the use of Finnish non-gendered pronouns, as well as the use of English gendered pronouns (Fukumura et al., 2013). Although such findings were taken to support the non-linguistic competition account, which claims that human referents of the same gender are more similar than those of different gender and pronoun use is sensitive to the referents’ similarity, we do not know if the effect generalises to any pronoun use. Antón-Méndez (2010) found that native speakers of Spanish, a language that allows null pronouns like Italian, were more prone to gender selection errors when producing *she* or *he* in English as their second language (L2) than L2 French speakers of English. Antón-Méndez argued that Spanish L2 speakers of English sometimes failed to include the referent’s gender feature in their preverbal message when speaking in English, because their L1 (Spanish) allows null pronouns, unlike French. Antón-Méndez’s proposal was supported by more recent computational modelling by Tsoukala, Frank, & Broersma (2017), showing that a null-pronoun feature in L1 increased the probability of gender selection errors in L2. Hence, although the use of non-gendered overt pronouns can be affected by the referents’ gender congruence, this might not be the case with null pronouns: Gender congruence between human referents may not affect non-linguistic competition for the use of null pronouns.

Unlike English or Finnish, Italian has *grammatical gender* (Corbett, 1991). Although gender congruence between human referents can be determined non-linguistically, grammatical gender can only be represented linguistically as it is a word’s syntactic property (Caramazza, 1997; Levelt et al., 1999; Schriefers, 1993; Vigliocco, Antonini, & Garrett, 1997). Cubelli, Lotto, Paolieri, Girelli, & Job (2005) reported that the production of bare nouns (i.e., nouns without any agreeing determiner or modifier) in Italian was delayed when distractor words had the same grammatical gender than when it had different grammatical gender as the target noun. This indicates that grammatical gender congruence between the lexical candidates enhances competition because same-gender lexical alternatives compete more than different-gender alternatives. Although Experiments 1 and 2 showed that the shared semantic categories or phonological properties between the antecedents are unlikely to influence pronoun use, fewer null pronouns in response to the antecedent nouns’ grammatical gender congruence would provide support for the linguistic competition account. On the contrary, the absence of the grammatical gender congruence effect in the use of null pronouns would further bolster the non-linguistic competition account.

**Figure 5.**
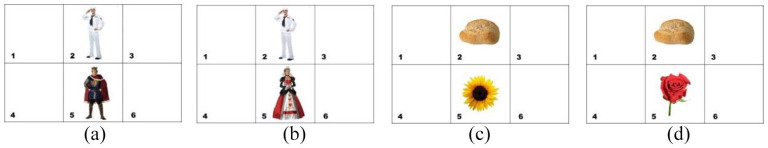
Example displays in Experiment 3. Human referents: (a) same gender and (b) different gender. Inanimate referents: (c) same gender and (d) different gender. Sailor: © California Costume Collections, Inc. King & Queen: Costume images used by permission In Character Costumes. Division of Fun World Easter Unlimited Inc. All Rights Reserved.

Experiment 3 thus manipulated the referents’ gender congruence for inanimate referents as well as for human referents. The experiment was also conducted in English to avoid null results (in case neither gender congruence affects the use of null pronouns in Italian). Speakers of Italian or English referred to human referents (5a and 5b, Figure 5a and b) or inanimate referents (5c and 5d, Figure 5c and d), and the referential candidates had either the same gender (5a and 5c, Figure 5a and c) or different genders (5b and 5d, Figure 5b and d). English has no grammatical gender, so the absence of a gender congruence effect in English would ensure that the grammatical gender effect in Italian resulted from the shared grammatical gender in Italian, rather than idiosyncratic non-linguistic differences between the inanimate entities.

**Table:** 

Experiment 3A: Italian	Experiment 3B: English
5. Context sentences	
(a) Il marinaio sopra il re è sul numero 2.	The sailor above the king is on Number 2.
(b) Il marinaio sopra la regina è sul numero 2.	The sailor above the queen is on Number 2.
(c) Il pane sopra il girasole è sul numero 2.	The bread above the sunflower is on Number 2.
(d) Il pane sopra la rosa è sul numero 2.	The bread above the rose is on Number 2.
6. Target descriptions	
(e) Adesso {il marinaio / Ø} è sul numero 3.	Now {the sailor/he} is on Number 3.
(f) Adesso {il pane / Ø} è sul numero 3.	Now {the bread/it} is on Number 3.

### Method

#### Participants

A total of 32 native speakers of Italian and 32 native speakers of British English were recruited from the University of Milano-Bicocca and the University of Stirling student community, respectively, in exchange of course credits or cash.

#### Materials

We used 40 experimental items, and these were mostly the same as those used in Fukumura, Pozniak, & Alario (2021). In each item, the referential candidates were either both human (5a and 5b) or both inanimate (5c and 5d). Within each referent condition, the referents had either the same gender (5a and 5c) or different genders (5b and 5d) in Italian. Figure 5 shows examples of the displays. We used 20 male and 20 female human characters as well as 20 masculine and 20 feminine inanimate objects. Each character or object was used as a target referent and as a same-gender competitor and a different-gender competitor across two different items (i.e., the competitor attributes were counterbalanced across conditions). The competitor pair for each target had comparable roles (boy and girl) or professions (king and queen) or related categories (e.g., both competitors were flowers), but their role or category was clearly distinguishable from the target’s. The conditions were distributed such that each participant saw each object/character once as a target and once as a competitor. The materials were the same for the Italian and the English experiments, except for two human characters (*majorette* and *dame* in Italian were replaced by cheerleader and duchess in English). In addition, we used 80 filler items, similar to those used in Experiments 1 and 2.

#### Design

We used a 2 (referent: human vs. inanimate) × 2 (gender congruence: same vs. different) repeated measures design. Together with 80 filler items, 40 experimental items were distributed across four lists in a fixed quasi-random order, subject to the constraint that the same target or competitor gender should not occur in more than three experimental trials consecutively and there should be at least one filler item between the experimental trials. Each list contained 10 items in each condition, with only one version of each item. Eight participants were randomly assigned to each list.

#### Scoring

We scored whether participants produced a null pronoun (*n* *=* 582) or a repeated noun (*n* *=* 675) in Italian (Experiment 3A) or whether they used a (overt) pronoun (*n* *=* 362) or repeated noun (*n* *=* 877) in English (Experiment 3B). As before, overt pronouns (*n* *=* 14) were rare in Italian, which were excluded from analyses. Other cases were excluded from analyses when participants referred to the competitor rather than the target referent (*n* *=* 3 in Experiment 3A; *n* *=* 4 in Experiment 3B); they changed their initial responses (*n* *=* 5 in Experiment 3A; *n* *=* 1 in Experiment 3B) or used a non-repeated noun (*n* = 1 I Experiment 3A); there was a technical error (*n* *=* 3 in Experiment 3B). Additional cases were excluded from Experiment 3B (English), where participants used “it” for human targets (*n* *=* 1) or marked a wrong gender on the pronoun (*n* *=* 18) or they did not start a new sentence (e.g., and has moved to Number 1) or dropped the subject or only mentioned the location (e.g., Now on Number 6; *n* *=* 14). In total, we excluded 23 cases (1.8% of total responses) and 41 cases (3.2% of total responses) from Experiments 3A and 3B, respectively.

### Results

**Figure 6.**
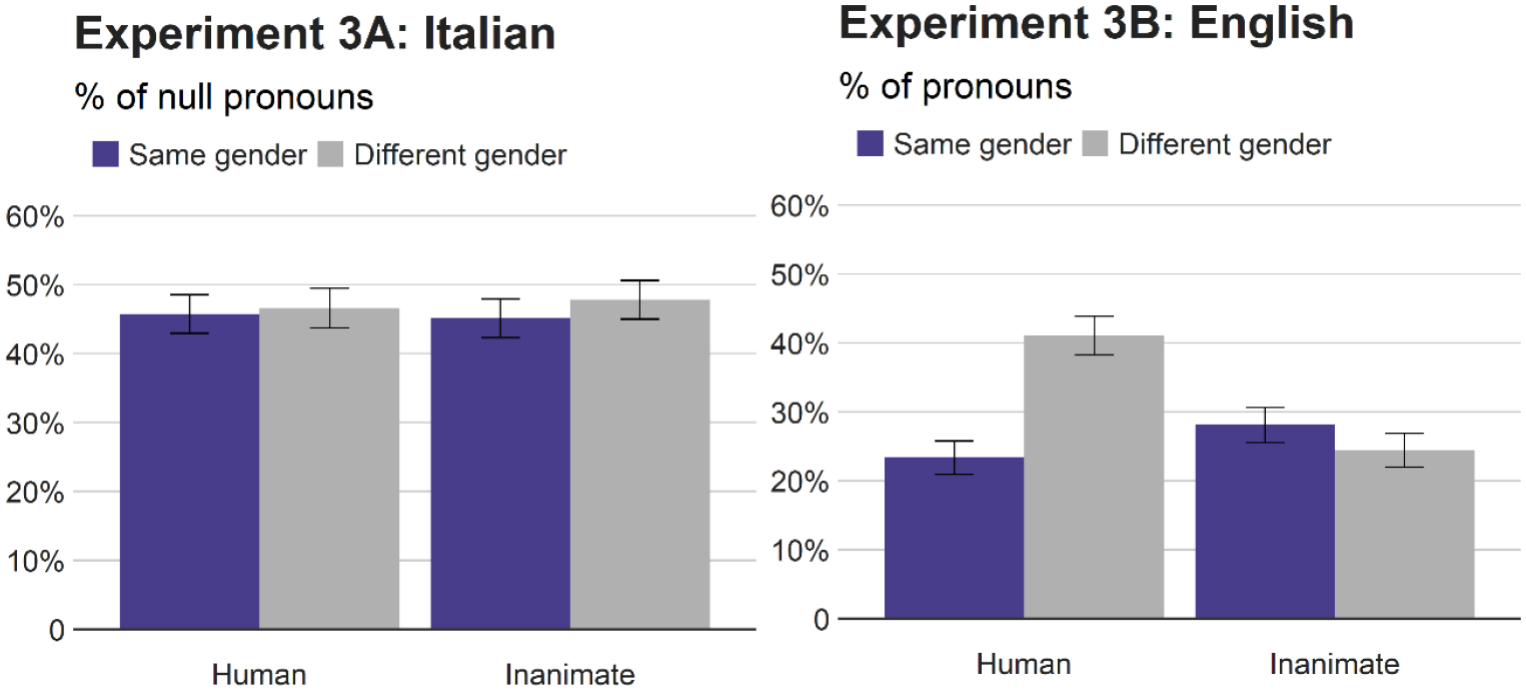
Percentages of null pronouns (Italian) and pronouns (English) relative to repeated nouns (Experiment 3). Error bars represent standard errors.

Figure 6 reports the means. The logit mixed effect analyses were carried out as before, including referent (human vs. inanimate) and gender (congruent vs. incongruent) as fixed effects. Table 7 summarises the results.

**Table 7.** Analyses on the choice of expressions in Experiment 3.

	Estimate	*SE*	*z*	*p*
Italian (Experiment 3A)				
(Intercept)	−0.49	0.68	−0.73	.468
Gender congruence	−0.08	0.09	−0.95	.344
Referent (human vs. inanimate)	−0.01	0.09	−0.16	.871
Gender congruence × Referent	0.04	0.09	0.49	.623
English (Experiment 3B)				
(Intercept)	−1.58	0.40	−3.95	<.001*
Gender congruence	−0.24	0.10	−2.51	.012*
Referent	0.29	0.09	3.27	.001*
Gender congruence × Referent	−0.43	0.10	−4.17	<.001*
Simple effects				
(Intercept)	−1.25	0.41	−3.08	.002*
Gender congruence for humans	−0.69	0.17	−4.06	<.001*
(Intercept)	−1.72	0.38	−4.52	<.001*
Gender congruence for inanimates	0.16	0.11	1.42	.155
Combined analyses				
(Intercept)	−1.05	0.38	−2.77	.006*
Language	0.63	0.37	1.69	.090
Gender congruence	−0.16	0.06	−2.64	.008*
Referent	0.13	0.06	2.21	.027*
Language × Gender congruence	0.08	0.06	1.32	.187
Language × Referent	−0.15	0.06	−2.50	.012*
Gender congruence × Referent	−0.19	0.07	−2.65	.008*
Language × Gender congruence × Referent	0.23	0.06	3.90	<.001*

*SE*: standard error. *Significance level α = 0.05.

In Italian, the analyses found no main effect of gender congruence, reflecting the similar rates of null pronouns in the same gender condition (45%) and in the different gender (47%) condition. Similarly, the rates of null pronouns were unaffected by whether the referents were human (46%) or inanimate (46%), and there was no significant interaction. By contrast, a significant main effect of referent was found in the use of English pronouns, with significantly more pronouns for human referents (32%) than for inanimate referents (26%). In English, the main effect of gender congruence and the gender congruence × referent interaction were also significant. Simple effects revealed that gender congruence reduced the rates of pronouns significantly for human referents (18%), whereas grammatical gender congruence present in Italian had no effect on the use of pronouns. The combined analyses confirmed a significant language × referent interaction as well as a significant language × gender congruence × referent interaction.

### Discussion

Speakers of English used fewer pronouns when the human referents share the same gender than otherwise, as demonstrated previously (e.g., Arnold & Griffin, 2007; Fukumura et al., 2010), whereas grammatical gender congruence between the inanimate referents, specified for Italian, did not alter the rates of English pronouns. In Italian, neither grammatical gender congruence between the inanimate referents nor gender congruence between the human referents affected the use of null pronouns. The absence of the grammatical gender congruence effect in Italian was in keeping with the non-linguistic competition account, and the absence of the human gender congruence effect in the use of Italian null pronouns provided support for the hypothesis that speakers do not take account of the referent’s gender information for the use of null pronouns (Antón-Méndez, 2010; Tsoukala et al., 2017).

## Experiment 4

The results of Experiment 3 ran counter to some linguistic theories that consider null pronouns in languages such as Italian as being the unpronounced version of overt pronouns (D’Alessandro, 2015; Rizzi, 1982, 1986). According to these theories, when a null pronoun refers to an antecedent in the discourse, it carries all the grammatical features of its antecedent (e.g., number, case, and agreement) similar to a full pronoun or noun. In the case of Italian, these features are visible in the inflections for gender and/or number of an adjective or a past participle. Moreover, researchers have argued that verb inflections are a form of referring expression (Ariel, 2000; cf. Givón, 1976), derived from anaphoric pronouns. In Experiment 3, participants mostly used a simple present tense that did not specify the gender of the referent (e.g., *Adesso è sul 3*; Now is on Number 3). Hence, gender congruence between the referents might not have affected the null pronoun rates, because speakers did not mark gender in the utterance. That is, speakers might take account of the referents’ gender information only when they produce gender-marked utterances. Although this contrasts with Fukumura et al. (2013) who argued that gender is one of the attributes speakers take account of regardless of whether the pronoun marks gender, there is good reason to believe that the verb’s gender agreement is critical for the use of null pronouns: The verb’s gender identifies the referent uniquely when the referents have different genders, but not when the referents have the same gender. Thus speakers might use fewer null pronouns and more nouns in the same gender condition than in the different gender condition to avoid the gender ambiguity of the verb agreement.

Thus, in the fourth and final experiment, Italian speakers were asked to use present perfect tense (*Adesso* ∅*/il marinaio si è spostato sul numero 3*; Now he/the sailor has moved to Number 3), which marks the referent’s gender in the participle (*spostato* for males and *spostata* for females). To avoid null findings, the referents were either both in a red box (two-box condition) or only the target referent was in a red box (one-box condition) as in Experiments 1 and 2, and the experiment was also conducted in English. This also allowed us to examine whether speakers of English adapt their pronoun use, depending on whether other source of information disambiguates gender ambiguous pronouns. If the gender effect in English is driven by ambiguity avoidance, speakers should avoid gender ambiguous pronouns more in the two-box context, where the referential competitor could also be the referent, than in the one-box context, where the absence of the box for the competitor disambiguates the gender ambiguity of the pronoun.

### Method

#### Participants

Forty native speakers of Italian and 32 native speakers of British English were recruited from the same population as before. One Italian-speaking participant who always produced overt pronouns was replaced from the Italian data. Two English-speaking participants who produced “it” to refer to human characters or omitted the subject in most trials were also replaced.

#### Materials and procedure

We constructed 40 experimental items, each having two referential candidates, who were always human. We varied their gender congruence, as in Experiment 3. The referential candidates were either both in red boxes or only one of them (the target referent) was in a red box as in Experiments 1 and 2.

#### Design

We used a 2 (gender congruence: same vs. different) × 2 (situational congruence: one-box vs. two-box) repeated measures design for each language, resulting in the creation of four lists. The experimental items and 80 filler items were distributed across four lists as before, and 8 participants were randomly assigned to each list. The procedure was the same as before.

#### Scoring

As before, we scored whether participants produced null pronouns (*n* = 533) or repeated nouns (*n* = 1,007) in Italian (Experiment 4A) or pronouns (*n* = 476) or repeated nouns (*n* = 752) in English (Experiment 4B). Cases were excluded when participants changed responses (including tense in Italian; *n* = 6 in Experiment 4A; *n* = 6 in Experiment 4B); they used a different construction (*n* = 1 in Experiment 4A; *n* = 5 in Experiment 4B); they made a substitution error mentioning the competitor (*n* = 2 in Experiment 4B); they accidentally skipped trials (*n* = 8 in Experiment 4A); they produced overt pronouns in Italian (*n* = 2; Experiment 4A) or they used “it” (*n* = 32) or dropped the subject (*n* = 5) or produced an inaudible response (*n* = 1) in English (Experiment 4B). In addition, 43 trials were excluded from Italian when participants did not use the past participle tense. In total, 60 responses (4%) and 52 responses (4%) were excluded from Experiments 4A and 4B, respectively.

### Results

**Figure 7.**
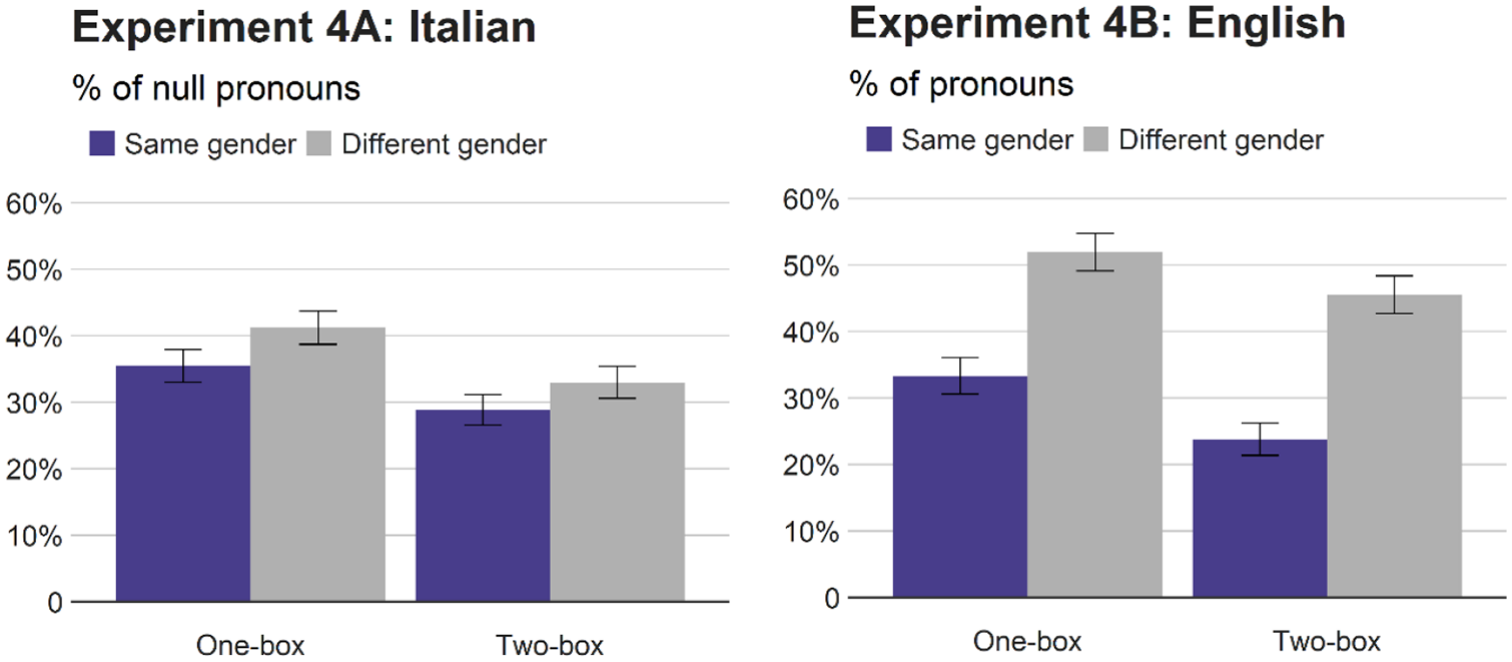
Percentages of null pronouns (Italian) and pronouns (English) relative to repeated nouns (Experiment 4). Error bars represent standard errors.

Figure 7 reports the means and Table 8 summarises the results. In Italian, the main effect of situational congruence indicated fewer null pronouns in the two-box condition (31%) than in the one-box condition (38%). Critically, the main effect of gender congruence was not reliable, and there was no significant situation × gender interaction. On the contrary, in English, speakers used fewer pronouns when the human referents had the same gender (29%) than when they had different genders (49%), and the referents’ situational congruence also affected the pronoun rates with fewer pronouns in the two-box context (35%) than in the one-box context (43%). The effect of gender congruence did not interact with the referents’ situational congruence, and the simple effects indicated that speakers used fewer pronouns when the referents had the same gender than when they had different genders, not only in the two-box context but also in the one-box context. The combined analyses confirmed these results, showing that although the effect of gender congruence interacted with language, the effect of situational congruence did not interact with language.

**Table 8.** Analyses on the choice of expressions in Experiment 4.

	Estimate	*SE*	*z*	*p*
Italian (Experiment 4A)				
(Intercept)	−2.37	0.75	−3.17	.002*
Gender congruence	−0.15	0.10	−1.40	.161
Situational congruence	−0.44	0.11	−3.95	<.001*
Gender congruence × Situational congruence	0.05	0.12	0.42	.676
English (Experiment 4B)				
(Intercept)	−1.42	0.67	−2.11	.035*
Gender congruence	−1.04	0.16	−6.43	<.001*
Situational congruence	−0.51	0.11	−4.74	<.001*
Gender congruence × Situational congruence	−0.11	0.10	−1.06	.290
Simple effects				
(Intercept)	−0.77	0.69	−1.12	.264
Gender congruence in the one-box context	−0.98	0.21	−4.59	<.001*
(Intercept)	−1.84	0.67	−2.72	.006*
Gender congruence in the two-box context	−1.15	0.18	−6.27	<.001*
Combined analysis				
(Intercept)	−1.93	0.50	−3.84	<.001*
Language	0.40	0.48	0.84	.404
Gender congruence	−0.54	0.10	−5.40	<.001*
Situational congruence	−0.47	0.08	−5.73	<.001*
Language × Gender congruence	−0.45	0.09	−5.09	<.001*
Language × Situational congruence	−0.03	0.08	−0.38	.703
Gender × Situational congruence	−0.03	0.07	−−0.40	.692
Language × Gender congruence × Situational congruence	−0.07	0.08	−0.86	.391

*SE*: standard error. *Significance level α = 0.05.

### Discussion

As in Experiments 1 and 2, the referents’ situational congruence reduced the rates of both Italian null pronouns and English pronouns, whereas the referents’ gender congruence reduced the pronouns rates only in English. Unlike in Experiment 3, in Italian, gender-marking on the verb identified the referent uniquely in the different gender condition, but not in the same gender condition. Hence, if the effect of gender congruence on pronoun use was driven by gender ambiguity avoidance in the utterance, speakers should have used fewer null pronouns in the same gender condition than in the different gender condition. But this is not what we found; the rates of Italian null pronouns did not differ reliably, depending on whether the gender marked on the verb identified the referent uniquely or not. Furthermore, in English, the referents’ gender congruence did not interact with their situational congruence: Speakers used fewer pronouns in the same gender condition than in the different gender condition, not only when the referential competitor could also be the referent so the context did not disambiguate the ambiguity of the pronoun, but also when only the target referent could be the referent, so the context disambiguated the pronoun. Both results thus indicate that speakers do not choose the use of pronouns depending on whether other sources of information, such as the referential context and the gender marked on the verb, disambiguate pronouns.

## General discussion

We began this study by asking whether the use of pronouns is affected by the antecedents’ similarity as well as the referents’ similarity and if this is dependent on whether the pronouns are overtly realised. Experiment 1 showed that both Italian and English speakers use fewer pronouns when the referential alternative could also be the referent than otherwise, while the referents’ situational congruence did not delay the production of the repeated nouns. On the contrary, the antecedents’ semantic congruence delayed the onset latencies of the repeated nouns, but it did not result in fewer pronouns. Experiment 2 showed that the antecedents’ phonological similarity does not affect pronoun choice in either language, while it replicated the effect of the referents’ situational congruence in both languages. These findings provided support for the non-linguistic competition account: Speakers decide whether to use a pronoun or noun at a non-linguistic level, so their pronoun choice is affected by the referents’ non-linguistic competition rather than the antecedents’ lexical competition. Hence, while both Italian null pronouns and English pronouns are sensitive to the non-competition between the referents arising from their situational congruence, lexical competition between the antecedent nouns, arising from their semantic category congruence or their grammatical and phonological similarities, have no effect on pronoun use.

As discussed earlier, previous research has shown that speakers use fewer (overt) pronouns when the referential candidates share the same animacy (e.g., both human) or when the human referents share the same gender, compared with when they differ in animacy or gender. What follows from the current findings is that although animacy congruence and gender congruence between human referents might affect lexical competition because of the shared semantic features, they affect pronoun choice, not because they affect lexical competition, but rather because they increase competition between the referents at a non-linguistic level. If the choice of using a pronoun or a noun was sensitive to lexical competition between the antecedent nouns, our participants should have used fewer pronouns when the antecedent nouns shared the same semantic categories than otherwise; in this study, the referents’ semantic category congruence did delay the production of the repeated nouns, demonstrating an effect of lexical competition. Nevertheless, the referents’ semantic category congruence did not affect the rates of pronouns. By contrast, the referents’ situational congruence led to fewer pronouns in this study. Although this variable delayed the processing times for the referential context and occasionally the verbal response initiation times, it did not delay the production of the repeated nouns.

The findings may appear to be in contrast with some existing models of pronoun production. Schmitt et al. (1999) proposed that pronoun production in German involves access to the antecedent’s phonological as well as grammatical representation. In their model, speakers decide whether to use a pronoun or noun depending on the information status of the lexical concept of the antecedent noun, and pronoun production processes involve similar representations as the antecedent nouns. Jescheniak et al. (2001), however, argued that pronoun production involves access to the antecedents’ grammatical properties, but their phonology remains inactive. In their study, the production of pronouns was delayed when participants heard a distractor word that was semantically related to the antecedent noun, but the distractor word’s phonological similarity to the antecedent’s noun did not. Furthermore, unlike Schmitt et al. and Jescheniak et al., Meyer and Bock (1999) argued that speakers access the grammatical gender of the antecedent by accessing the preceding discourse (rather than accessing the antecedent noun’s lexical concept first). They reported that speakers of Dutch make fewer gender selection errors (e.g., using the common-gender pronoun *die* for a neuter-gender referent) when the referential alternative mentioned in the preceding sentence shares the same grammatical gender as the referent than otherwise. The current results do not distinguish these accounts. Critically, although speakers might access the antecedents’ linguistic representations such as semantic, grammatical or phonological features during pronoun production, such information is unlikely to influence the choice of using a pronoun or noun; this choice is made at an earlier stage of the language production process, driven by the referents’ non-linguistic representations.

The current results have implications for the issue concerning ambiguity avoidance. Specifically, Fukumura et al. (in press) reported that speakers of French consistently reduced pronoun use when human referents had the same gender and hence the use of gendered pronouns was gender ambiguous than when they had different genders, while they mostly failed to do so when non-human referents had the same grammatical gender, so the use of gendered pronouns was equally ambiguous. The current findings indicate that speakers choose whether to use a pronoun or noun at a non-linguistic level; although the antecedents’ grammatical gender is required for selecting pronominal forms at the lexical level, speakers decide whether to use a pronoun or a noun before accessing this information. Hence, grammatical gender congruence between the antecedent nouns is unlikely to influence the pronoun rates. In the series of experiments in French, speakers reduced the use of grammatical gender ambiguous pronouns only in one experiment, where the antecedents’ semantic category congruence was also varied. The experiments in this study showed that semantic category congruence alone does not affect pronoun use. The referents’ semantic category congruence affected the use of pronouns in French, most likely because it helped speakers avoid grammatical gender ambiguous pronouns, not because speakers generally used fewer pronouns when the referents were categorically related than otherwise.

Although research has assumed that gender congruence between human referents affects similarity-based competition (Arnold & Griffin, 2007; Fukumura et al., 2011; Griffin & Wangerman, 2013), our findings indicate that this does not necessarily hold for the use of null pronouns. In Experiment 3, while speakers of English produced fewer pronouns when the human referents shared the same gender than otherwise, the use of Italian null pronouns was affected by neither grammatical gender congruence between inanimate referents nor gender congruence between human referents. The referents’ gender congruence did not affect the use of Italian null pronouns even when the verb participle marked the referent’s gender so it determined the gender ambiguity of the utterance (Experiment 4). Hence, although speakers of Italian clearly take account of the gender information for the verb inflections, the gender information does not underlie the use of null pronouns, unlike the use of English pronouns. The results are thus clearly in contrast with the gender congruence effect for the use of Finnish non-gendered pronouns, whereby speakers of Finnish were reported to use fewer non-gendered pronouns when the human referents shared the same gender than not (Fukumura et al., 2013). Importantly, the rates of null pronouns in Italian were also unaffected by the humanness of the referent: In Experiment 3, although English speakers used more pronouns for referring to the human referents than for inanimate referents, humanness did not affect the use of Italian null pronouns. Note that the effect of humanness in English is unlikely to have arisen from the difference in the pronominal forms for human (*he, she*) and inanimate (it) referents. Speakers of French have also been found to use more third-person singular pronouns (*il*, *elle*) when referring to human referents than when referring to inanimate referents (Fukumura et al., 2020) and research has also shown that English speakers use the plural pronoun “they” more for plural human entities than for plural inanimate entities (Fukumura & Van Gompel, 2011).

We thus argue that the referents’ gender congruence and humanness are unlikely to affect the use of null pronouns because speakers tend not to attend to these features for the use of null pronouns during conceptual encoding. The results from the study by Van der Meulen, Meyer, & Levelt (2001) support this possibility. In their study, speakers looked at the referent less often and for a shorter duration when producing pronouns compared with when producing repeated nouns. Assuming that eye gaze reflects not only the speaker’s referential intentions but also the underlying lexical planning (Griffin, 2004; Meyer, Sleiderink, & Levelt, 1998), speakers should look at the referent less and for a shorter duration when producing null pronouns than when producing overt pronouns, because null pronouns do not even require lexicalisation. Hence, the representation that underlies null pronouns may be even more under-specified than overt pronouns, because speakers are less likely to attend to the referent’s features when the language allows null pronouns (compared with when it does not), and this is why the referents’ gender congruence is less likely to affect the use of null pronouns. On the contrary, the referents’ situational congruence affects the use of both overt pronouns and null pronouns, because this is a contextual variable that affects competition regardless of whether pronouns are lexicalised. Similarly, the antecedent’s grammatical role has been shown to influence the use of both null pronouns in Italian and overt pronouns in English. Presumably, the antecedent’s grammatical role affects the referent’s accessibility, regardless of whether the to-be-produced pronoun is overtly realised, such that speakers tend to use more null pronouns in Italian and more pronouns in English when the referent has been mentioned in the subject position than in another position.

One may, however, ask whether null pronouns were unaffected by the referents’ gender congruence because the antecedent was always the grammatical subject and the preference of null pronouns for subject antecedents is stronger than the preference for subject antecedents in English pronouns. That is, when the antecedents are the grammatical subject, Italian null pronouns may generally be much more preferred than alternative referring expressions than English pronouns are; hence speakers ignore the referents’ gender congruence for the use of null pronouns, assuming that the addressee should be able to identify the referent based on the antecedent’s grammatical role. In this study, the rates of Italian null pronouns and English pronouns did not differ reliably, however; hence, there was no indication that the subject preference was stronger for null pronouns in Italian than for pronouns in English. Also, if the use of null pronouns in Italian is more strongly constrained by the antecedent’s grammatical role than the use of pronouns in English, the referents’ situational congruence should have affected Italian null pronouns less than English pronouns. But this is not what we found. Although there was a marginal tendency for a stronger situational congruence effect in English than in Italian in Experiment 1, the interaction was non-significant in Experiments 2 and 4. Hence, null pronouns are unaffected by the referents’ gender congruence, not because null pronouns are insensitive to the referents’ competition in general, but rather because the referents’ gender congruence does not affect competition unless speakers initiate conceptual encoding for lexicalisation.

To conclude, the use of null pronouns as well as overt pronouns is sensitive to the referents’ non-linguistic competition; speakers use pronouns less often when the referential candidates compete more strongly non-linguistically, but they do not take account of the similarity of the antecedent nouns. Importantly, we also showed that what similarity affects pronoun use is partly dependent on the type of pronouns: The use of null pronouns is insensitive to gender congruence between the human referents, and we have argued that the representations underlying null pronouns are more underspecified than those for overt pronouns, so the use of null pronouns is less likely to be affected by the referents’ similarity.

## Notes

1. As explained in the material section, Italian and English had different unrelated competitors.
